# Updates in Intravenous Thrombolysis Treatment for Acute Ischemic Stroke in Asian Populations

**DOI:** 10.14336/AD.2025.0060

**Published:** 2026-04-12

**Authors:** Rui Xue, Anni Wang, Ying Zhou, Min Lou

**Affiliations:** ^1^Department of Neurology, the Second Affiliated Hospital, Zhejiang University School of Medicine, Hangzhou, China.; ^2^State Key Laboratory of Transvascular Implantation Devices, Hangzhou, China

**Keywords:** acute ischemic stroke, intravenous thrombolysis, Asian populations, treatment progress

## Abstract

Acute ischemic stroke (AIS) in Asian populations presents unique epidemiological characteristics and challenges, which are of great significance for grasping the effect of intravenous thrombolysis (IVT) in the treatment process. IVT has proven efficacy in improving outcomes for AIS patients, but its application in Asia faces distinct barriers, such as delays in diagnosis, differences in healthcare infrastructure, and variations in patient characteristics. This review examines recent research on the use of IVT for AIS in Asia, comparing findings with Western populations to identify both commonalities and disparities. We explore the current state of clinical practices, the challenges associated with treatment protocols, and the potential benefits of tailored therapeutic strategies. Additionally, the review highlights emerging trends and offers insights into how these factors may influence future research and clinical decision-making in the region.

## 1. Introduction

Stroke remains one of the leading causes of mortality and disability globally. As of 2021, it was the third leading cause of death and the fourth major contributor to disability-adjusted life years (DALYs) [1]. Acute ischemic stroke (AIS) accounts for 65.3% of incident strokes [1] and can be classified into five etiological subtypes: large-artery atherosclerosis (LAA), cardio-embolism, small-vessel occlusion, stroke of other determined etiology, and stroke of undetermined etiology [2]. According to the Global Burden of Disease (GBD) 2021 study, AIS has a profound global impact, with nearly 70 million cases globally, with an age-standardized prevalence of 819.5 per 100,000 individuals, a death rate of 44.2 per 100,000, and a DALYs rate of 837.4 per 100,000 individuals [3].

In China, it was reported that 73.2% of stroke survivors experienced cognitive impairment of Mini-Mental State Examination < 20 and 27% experienced severe disability of modified Rankin Scale (mRS) ≥ 3 at 3 months [4, 5]. Additionally, AIS patients are at heightened risk for acute myocardial infarction (AMI), which significantly increases in-hospital mortality rates [6]. These poor functional outcomes result from the brain's vulnerability to ischemic damage, even transient ischemia can trigger a series of events leading to neuronal cell death [7]. However, brain damage after AIS can be mitigated by preserving the "ischemic penumbra," the region of brain tissue that is severely hypoperfused and hypoxic but electrically silent and at risk of irreversible injury [8].

Given this pathophysiology, the early restoration of blood flow is crucial for improving outcomes. Evidence-based treatments like intravenous thrombolysis (IVT) and endovascular therapy focus on salvaging the penumbra by re-establishing cerebral perfusion as quickly as possible [9]. The concept of IVT was inspired by similar strategies used in the treatment of AMI, leading to major clinical trials for AIS beginning in the 1990s. These trials confirmed the efficacy of IVT in enhancing clinical outcomes, making it the standard of care for AIS in numerous international guidelines.

## 2. Characteristics of AIS in Asian Populations

### 2.1 AIS Burden in Asia

The global burden of AIS has shown significant regional variations, with Asia warranting particular attention, as the burden of AIS in Asia remains higher than that in Western countries [3]. In 2021, certain Asian subregions reported higher age-standardized incidence rates, mortality rates, and DALYs rates than both high-income North America and Western Europe.

### 2.2 Risk Factors and Etiology of AIS in Asia

The primary risk factors and etiological patterns of AIS in Asian populations differ from those in non-Asian groups. Hypertension, a key risk factor for AIS, doubles the risk for Asians compared to non-Asian populations [10]. Smoking, a well-established risk factor for AIS, has a higher prevalence among Asian males, particularly in South East Asia (68% in South Korea, 63% in China, and 47% in Japan), compared to the Western Pacific region [11]. Dyslipidemia, characterized by low HDL cholesterol and elevated triglycerides, is more prevalent in Asian populations and correlates with increased stroke risk, while some studies indicate that reduced LDL cholesterol levels may be associated with decreased stroke risk in certain population [12, 13]. Intracranial atherosclerotic disease demonstrates greater prevalence in Asians compared to whites [14]. For example, LAA is present in 25.4% of Chinese Stroke patients, compared to 14.7% in Caucasians. Additionally, Chinese populations reveal a higher proportion of small-vessel disease stroke (33.1% vs 19.3%) and a lower proportion of cardioembolic (15.8% vs 25.7%) and other/unknown ischemic stroke (23.2% vs 38.8% ) compared to European populations [15]. These differences in stroke subtypes are consistent across other Asian countries like Japan, Korea, and Thailand.

### 2.3 Functional Outcomes and Recurrence among Asian Populations

In contrast to short-term outcomes, long-term prognosis for Asians with AIS is often more favorable. A retrospective analysis of Asian American and white patients participated in the Get With The Guidelines-Stroke (GWTG-Stroke) program revealed that Asian American patients had worse in-hospital functional outcomes including higher in-hospital mortality, longer hospital stays, and reduced likelihood of independent ambulation at discharge than white patients [16]. However, this pattern reversed after adjustment for the National Institutes of Health Stroke Scale (NIHSS) scores. The international, Enhanced Control of Hypertension and Thrombolysis Stroke study (ENCHANTED) investigating clinical outcome disparities between Asian and non-Asian participants found no significant differences in functional outcome (defined by mRS scores 2-6 or 3-5) or death, despite Asians showing greater odds of intracerebral hemorrhage and neurological deterioration within 24 hours [17]. Additionally, a study focusing on patients aged over 65 years across 926 U.S. hospitals showed that Asian American patients had lower unadjusted mortality rates at both 30 days and 1-year post-stroke when compared to white patients (11.1% vs. 15.0% and 23.9% vs. 31.7%), with this difference persisting after adjustment [18]. These findings can likely be attributed to the greater incidence of small vessel lacunar strokes and intracranial atherosclerotic strokes among Asians, in contrast to the larger prevalence of cardioembolic strokes seen in white patients [15, 19]. A subanalysis of the TIAregistry.org project reported the 5-year risk of vascular events in Asian patients was as low as that in non-Asian patients (14.0% vs 11.7%; hazard ratio [HR], 1.10; 95% confidence interval [CI], 0.88-1.37; P = 0.41), while Asian participants exhibited a 2.2-fold higher risk of intracranial hemorrhage (1.8% vs 0.8%; HR, 2.23; 95% CI, 1.09-4.57; P = 0.029) [20].

Stroke recurrence rates also differ significantly between Asian and non-Asian populations. Research utilizing health data from Ontario, Canada, revealed that South Asian patients with diabetes experienced a 20% greater likelihood of stroke recurrence compared to non-South Asian individuals, despite being younger and more compliant with preventive medication at the start of the study [21]. This suggests that, while preventive measures may be well adhered to, underlying genetic or socioeconomic factors could contribute to the elevated recurrence risk among South Asians, highlighting the need for tailored approaches to stroke care and prevention across different ethnic groups.

## 3. Mechanisms and Innovations in Intravenous Thrombolysis

### 3.1 Thrombolytic Agents and its Mechanisms

Working by dissolving thrombi that obstructs cerebral blood flow, thrombolytic agents can be classified into three generations based on their mechanisms, efficacy, and safety profiles. While alteplase remains the gold standard, newer agents are poised to enhance AIS treatment by offering faster, more effective, and safer administration protocols.

#### 3.1.1 First-Generation Thrombolytic Agents: Streptokinase and Urokinase

These agents activate plasminogen into plasmin, an enzyme that degrades fibrin in thrombi. Both agents belong to non-specific plasminogen activators and carry higher risks of systemic bleeding and re-thrombosis. Streptokinase is a proteolytic enzyme obtained from hemolytic streptococcus, known for its lower cost and widespread use, has lower fibrin specificity and may provoke allergic reactions due to its antigenicity. Urokinase is a serine protease, which has a similar curative effect to streptokinase but has fewer allergic reactions and hypotensive reactions.

#### 3.1.2 Second-Generation Thrombolytic Agents: Alteplase, Anistreplase and Prourokinase

These agents have higher fibrin specificity and recanalization rate. The thrombolytic effect is better than that of the first-generation agents. It has fewer adverse reactions, and the occurrence of bleeding complications is relatively low as well. Alteplase (rt-PA) has higher fibrin specificity compared to streptokinase, improving its ability to selectively target thrombi. It is the standard of care for AIS but may cause mild fibrinogen depletion. Anistreplase, an anisoylated plasminogen/streptokinase activator complex (APSAC), has similar limitations to streptokinase, including high antigenicity. Prourokinase, the precursor to urokinase, is activated on the fibrin surface and has the potential to induce fibrin-specific lysis with fewer risks of re-thrombosis and bleeding [22]. A novel Recombinant Human Prourokinase (rhPro-UK) carries a safer profile, with minimal re-thrombosis risk and no procoagulant effects [23].

#### 3.1.3 Third-Generation Thrombolytic Agents: Reteplase and Tenecteplase

The emergence of third-generation thrombolytic agents was prompted by the drawbacks of second-generation agents, including their short half-life, high cost and high doses administered over a short period. These medications possess a longer half-life, increased specificity of fibrinolytic proteins, minimal depletion of fibrinogen, and demonstrate a remarkable thrombolytic effect on longer-forming thrombi. Reteplase, a non-glycosylated variant of rt-PA, exhibits an extended half-life and can be delivered via a double-bolus regimen [24]. Tenecteplase, a genetically modified rt-PA, represents a promising alternative IV thrombolytic agent offering several advantages over rt-PA [25], including improved fibrin specificity, an extended half-life, and the convenience through a single IV bolus [26].

### 3.2 Historical Development of Intravenous Thrombolysis: from Time Window to Tissue Window

The development of IVT has been significantly shaped by the understanding of ischemic penumbra and the concept of a therapeutic time window. Early research, including Astrup's penumbra theory in 1977, showed that ischemic penumbra tissue, which is at risk but not yet irreversibly damaged, could be salvaged with timely reperfusion. This laid the groundwork for thrombolytic therapies targeting ischemic but viable brain tissue.

The landmark NINDS rt-PA Stroke Study (1995) established a 3-hour time window for intravenous rt-PA administration, and the ECASS III trial (2008) extended this window to 4.5 hours. However, as imaging technology advanced, it became apparent that the "time" approach had limitations. The WAKE-UP trial (2018) and EXTEND trial (2019) introduced the concept of the "tissue window", demonstrating that salvageable brain tissue could exist beyond the standard time window by using advanced imaging techniques, such as CT and MRI perfusion, to visualize the ischemic penumbra. The tissue window concept is actually based on the biological evolution of ischemic injury. Brain ischemia is a dynamic process, and the tolerance of brain tissue to ischemic damage varies across patients due to differences in cerebral reserve, collateral circulation, infarct size, and the completeness of tissue damage [27]. This variability means that ischemic tissue may remain viable beyond the traditional time window, particularly in patients with robust collateral circulation or slow infarct progression.

### 3.3 Clinical Application of Tissue Window in Asian Populations

The tissue window concept has particular relevance in Asian populations, where differences in stroke etiology, collateral circulation, and response to treatment have been observed. The THAWS trial conducted in Japan investigated the application of intravenous rt-PA in patients whose last-known-well time exceeded 4.5 hours but who met the eligibility criteria based on MRI findings, specifically the identification of acute ischemic lesions on diffusion-weighted imaging (DWI) without significant corresponding hyperintensity on fluid-attenuated inversion recovery (FLAIR) [28]. The ROSE-TNK and TRACE-III trials conducted in China were instrumental in extending the thrombolysis treatment window to 24 hours for AIS patients. These trials used advanced imaging techniques, including DWI-FLAIR mismatch and CT/MRI perfusion imaging, to assess brain tissue viability [29, 30]. A key criterion in TRACE-III trial was a ratio of the volume of hypoperfused tissue to the ischemic core volume of at least 1.8, indicating that ischemic tissue could still be rescued beyond the 4.5-hour window. Similarly, the CHABLIS-T trial in China extended the intravenous tenecteplase window to 24 hours, utilizing a favorable penumbral profile defined as a hypoperfusion lesion-to-infarct core volume ratio greater than 1.2 on computed tomography perfusion (CTP) [31]. The results of these studies reflect a growing understanding that ischemic tissue salvageability—rather than time—is the critical factor in determining whether thrombolysis should be administered. This personalized approach, especially when guided by favorable imaging profiles, has significant implications for patient outcomes, focusing on the physiological state of the brain rather than simply elapsed time. Our previous research has focused on determining optimal thresholds for ischemic core and penumbra using CTP and magnetic resonance perfusion (MRP), identifying regions where thrombolysis could be most beneficial [32, 33].

## 4. Specificities of Intravenous Thrombolysis in Asian Populations

### 4.1 Differences in Treatment Outcomes: Efficacy and Complications

Previous literature suggests that the overall efficacy of IVT between Asian and non-Asian populations is generally comparable. The VISTA randomized neuroprotection trials (250 Blacks, 112 Asians, and 3642 Caucasians) investigated whether non-Caucasian patients of diverse national backgrounds exhibited different responses to tissue plasminogen activator when compared to Caucasian patients. Data analysis revealed no significant racial or ethnic disparities in outcomes, as assessed by the ordinal mRS at 90 days, or dichotomized outcomes such as mRS 0-1, mRS 0-2, and survival [34]. Likewise, according to a comparative analysis of ENCHANTED study (2977 Asians and 1574 NonAsians), there was no significant differences in functional outcome defined by mRS 2-6 (47.7% vs. 54.7%) or death (8.0% vs. 11.2%) within 90 days between Asians and non-Asians, despite Asians having greater odds of intracerebral hemorrhage (18.8% vs. 18.0%) and neurological deterioration within 24h (9.2% vs. 7.6%). [17]. Moreover, participants from China in the ENCHANTED study exhibited relative higher 90-day mRS of 2-6 (55.6% vs. 47.8%; P = 0.20) compared to non-China participants, even though significant differences in demographic and risk factors [35]. Other large-scale studies directly comparing the IVT efficacy between Asian and non-Asian populations remain scarce to date.

Although meta-analysis suggests a similar risk of hemorrhage, larger-scale studies and higher-level evidence indicate that Asian AIS patients may face an elevated risk of symptomatic intracerebral hemorrhage (sICH) following IVT compared to non-Asian patients. A meta-analysis of IVT for AIS in Asia revealed that both dosing regimens of rt-PA thrombolysis in Asia were associated with rates of sICH similar to those observed in the NINDS (USA, 6.4%) and SITS-MOST (Europe, 4.6%) studies. Only one study using the standard dose (0.9 mg/kg) and one employing the low dose (0.6 mg/kg) reported unacceptably high rates of hemorrhagic complication following thrombolysis (23.8% and 14.5%, respectively) [36]. However, the GWTG-Stroke program found that Asian patients had a notably higher rate of sICH at 6.8%, compared to 4.8% in White patients, 4.1% in Black patients, and 4.7% in Hispanic patients, even after adjusting for baseline confounders [37]. The underlying reasons remain unclear. Regional differences in clinical practice and ethnic disparities are potential reasons. A previous study from the GWTG registry evaluating the predictors of sICH in AIS patients receiving rt-PA has shown Asian race (compared with non-Asian) to be associated with an independent risk of sICH (OR 2.12) [38]. Additionally, it has been proposed that the presence of diffuse atherosclerosis, more common in older Asian patients with higher comorbidity rates, could weaken blood vessel walls, increasing vascular permeability and facilitating red blood cell leakage across damaged vessels post-thrombolysis [39]. More large-scale, multi-center studies are needed to further clarify these differences and develop more personalized treatment strategies such as reduction in rt-PA dose for different populations.

### 4.2 Drug Selection: Comparing Thrombolytics in Asian and Non-Asian Populations

Overall, intravenous rt-PA has demonstrated efficacy and safety in treating AIS across both Asian and non-Asian populations. Several Asian studies have confirmed the benefits of intravenous rt-PA when administered within 4.5 hours of symptom onset. A comparative study by Zheng et al. found better 3-month outcomes in Chinese patients (mRS 0-1: 63.3%) than those in the ECASS III trial (mRS 0-1: 56.7%) treated with intravenous rt-PA [40], and a study from the SITS-NEW registry also indicated a greater proportion of Asian patients achieved a mRS score of 0-2 at 3 months (62.5%) compared to Europeans in the SITS-MOST trial (54.8%)[41].

Tenecteplase has gained recognition as a promising alternative to rt-PA for treating AIS in both Asian and non-Asian populations. The TRACE study, a multicenter, randomized, open-label, blinded-endpoint controlled phase II trial conducted in China, demonstrated that tenecteplase exhibited comparable safety outcomes to rt-PA at all tested doses (0.1, 0.25, and 0.32 mg/kg) when administered within 3 hours of symptom onset[42]. Building upon these findings, both TRACE-2 and ORIGINAL studies further compared intravenous tenecteplase (0.25 mg/kg) with rt-PA in patients eligible for treatment within 4.5 hours of stroke onset. Both studies showed that tenecteplase was non-inferior to rt-PA in terms of functional outcomes and safety [43, 44]. The TRACE-III, ROSE-TNK, and CHABLIS-T trials conducted in China extended the tenecteplase treatment window to 24 hours post-stroke onset, guided by salvageable brain tissue on imaging [29-31].

Nevertheless, ethnicity-specific differences in the efficacy and safety of tenecteplase have emerged in recent studies. A systematic review and meta-analysis investigating these differences found that tenecteplase was linked to significantly greater rates of complete recanalization but higher mortality rates and lower rates of mRS 0-2 in Asians compared to Caucasians [45]. The observed differences can likely be explained by a range of factors, such as genetic variations, stroke subtypes, and differences in patient selection. As such, there may be important clinical implications for selecting tenecteplase or rt-PA in different ethnic groups, and further research is needed to address these potential disparities.

Recently, two clinical trials conducted in China suggested that intravenous reteplase may offer similar or superior efficacy outcomes compared to rt-PA in AIS patients treated within 4.5 hours of symptom onset. Specifically, a phase 2, randomized, controlled trial found that the reteplase group (12+12 mg or 18+18 mg) exhibited a comparable profile of clinical efficacy outcomes to the standard rt-PA group, without a notable rise in the rate of sICH [46]. Furthermore, the RAISE trial compared 18+18 mg reteplase with rt-PA and found that reteplase was more likely to lead to an excellent functional outcome (90-day mRS 0-1: 79.5% VS 70.4%) [47]. However, there is a lack of studies on intravenous reteplase in non-Asian populations, and further research is needed in diverse populations.

### 4.3 Dose Adjustments: Is Lower Dosage Needed in Asian Populations?

Concerns have arisen regarding the safety of standard 0.9 mg/kg dose of intravenous rt-PA in Asian populations, despite its approval for AIS treatment in the U.S. back in 1996. Research indicates that there may be ethnic variations in coagulation-fibrinolysis factors, with evidence showing that Caucasians tend to have elevated plasma levels of fibrinogen and plasminogen activator inhibitors when compared to Japanese individuals [48, 49]. These differences have led to the investigation of lower-dose rt-PA as a potentially safer alternative in Asian populations.

The Japan Alteplase Clinical Trial (J-ACT) evaluated the efficacy and safety outcomes of AIS patients administered 0.6 mg/kg intravenous rt-PA within a 3-hour window after stroke onset, contrasting these outcomes with those from the NINDS trial's standard 0.9 mg/kg rt-PA. The results revealed comparable outcomes between the two groups: the 3-month mRS 0-1 was 36.9% for the low-dose group versus 39% for the standard-dose group, and the incidence of sICH was 5.8% versus 6.4%, respectively [50]. A pooled analysis combining data from nine Asian stroke registries further indicated that clinical outcomes with low-dose rt-PA were comparable to those with standard-dose rt-PA. The adjusted odds ratio for combined death or disability was 1.00 (0.85-1.19), and for sICH, it was 0.87 (0.63-1.19), with lower mortality approaching borderline significance at 0.77 (0.59-1.01) [51].

However, results from the ENCHANTED study showed that for thrombolysis-eligible AIS patients, low-dose rt-PA administration failed to achieve the predefined noninferiority margin of the conventional composite endpoint of death or disability (mRS 2-6 at 90 days) compared to the standard-dose rt-PA (53.2% in the low-dose group and 51.1% in the standard-dose group; OR, 1.09; 95% CI, 0.95 to 1.25; P = 0.51 for noninferiority). When evaluating functional outcome through ordinal mRS analysis, the low-dose rt-PA regimen demonstrated noninferiority compared to standard-dose, with an unadjusted common odds ratio of 1.00 (95%CI, 0.89-1.13; P = 0.04 for noninferiority), and resulted in significantly less severe sICH (1.0% in the low-dose group and 2.1% in the standard-dose group; P = 0.01) [52]. Secondary analysis of the ENCHANTED study also showed no significant divergence in the treatment effects between low and standard-dose rt-PA for either the combined endpoint of death and disability or the ordinal shift in the mRS at 90 days across different ethnicities. Statistically similar decreases in sICH rates with low-dose rt-PA were observed in both Asian and non-Asian groups [53].

While low-dose intravenous rt-PA may be safer, the optimal dosage for Asian populations remains challenging due to limited evidence from randomized controlled trials (RCTs). Consequently, selecting appropriate thrombolytic drug dosage should be individualized according to each patient's unique condition and the risk of adverse events.

### 4.4 Penumbra-Guided Thrombolysis: Impact of Collateral Circulation in Asia

The concept of penumbra-guided thrombolysis has become a focal point for extending the therapeutic window for IVT. Actually, penumbra and collateral circulation are closely linked, as well-developed collaterals help maintain perfusion in the penumbra, thereby reducing the volume of ischemic core tissue and minimizing neuronal damage [54]. This approach is increasingly relevant in both Asian and non-Asian populations, particularly as collateral circulation mechanisms may vary due to differences in vascular anatomy. In Asia, intracranial artery stenosis is more prevalent, and the presence of robust collateral circulation could potentially contribute to improved outcomes following thrombolysis. A key question arises: does the superior collateral circulation in Asian populations, possibly resulting from the higher incidence of intracranial artery stenosis, provide an added benefit for IVT, especially when guided by penumbra imaging?

Recent studies support the notion that the presence of penumbral tissue, identified through imaging, could extend the therapeutic window for IVT beyond the typical 4.5 hours. For example, the Chinese ROSE-TNK trial demonstrated favorable outcomes (52.5% achieving mRS 0-1) for individuals receiving intravenous tenecteplase within an extended 4.5-24 hour window who displayed DWI-FLAIR mismatch, suggesting that tenecteplase could represent a safe and effective therapeutic option for late-presenting patients with potentially salvageable brain tissue [29]. Furthermore, the CHABLIS-T trial conducted in China indicated that intravenous tenecteplase, administered within an extended 4.5 to 24-hour window, also demonstrated safety and efficacy in patients experiencing anterior circulation large/medium vessel occlusion or significant stenosis and salvageable tissue on perfusion imaging, underscoring the importance of assessing collateral circulation in this population [31].

On the other hand, research involving non-Asian populations, such as the TIMELESS trial conducted in U.S. and Canada, have shown that intravenous tenecteplase administration within a 4.5-24-hour window failed to improve functional outcomes when measured against placebo. While the results did not show definitive advantages, the rates of sICH and 90-day mortality were similar across both groups [55]. Similarly, the EXTEND trial showed that intravenous rt-PA administration within a 4.5-9 hour window after stroke onset resulted in higher favorable outcomes (90-day mRS 0-1: 35.4%) compared to placebo, but with an elevated incidence of sICH (6.2% vs. 0.9%) [56].

Apart from issues with the experimental design itself, the differences in outcomes between Asian and non-Asian populations may be partly attributed to the differences in collateral circulation. In Asia, the higher prevalence of intracranial artery stenosis may lead to more pronounced collateral circulation, which could improve perfusion to affected brain areas, especially when thrombolysis is administered to patients with salvageable tissue. This could, in turn, reduce the likelihood of poor outcomes despite delayed intervention. While there is still a need for further research to confirm these hypotheses, it is possible that collateral circulation plays a crucial role in improving thrombolytic therapy effectiveness in Asian populations, particularly in cases where the penumbra is present and therapeutic time window has been prolonged.

### 4.5 Blood Pressure Management in Intravenous Thrombolysis: Impact of Intracranial Artery Stenosis (ICAS)

Blood pressure (BP) management is a critical concern in Asian patients receiving IVT. Studies in India have shown that fluctuations in early systolic blood pressure (SBP) and high SBP levels correlate with heightened risks of sICH and 3-month poor outcomes following thrombolysis [57]. In contrast, maintaining stable or decreasing BP during the treatment period is linked to better functional outcomes [58]. Specifically, a Chinese study demonstrated that keeping SBP ≤148 mmHg during the first 24 hours after IVT, followed by a range of 127-138 mmHg, improves outcomes [59]. However, a Korean trial investigating intensive BP management (target SBP <120 mmHg) in ICAS patients did not show non-inferiority compared to a moderate approach (target SBP <140 mmHg), and the intensive control group experienced a larger ischemic lesion volume during the subacute phase of AIS [60]. Additionally, there is growing concern that overly aggressive BP lowering in ICAS patients may have unintended consequences. Specifically, low BP levels may elevate the likelihood of stroke recurrence among individuals presenting with bilateral internal carotid artery stenosis [61]. Thus, while there is a general benefit to controlling BP during the period of thrombolysis, further lowering of BP targets in ICAS patients requires cautious consideration due to the limited evidence available.

### 4.6 Special Situations for Intravenous Thrombolysis

#### 4.6.1 Mild Stroke

Mild strokes are particularly prevalent in Asia, especially in China, where approximately 30% of new strokes each year are classified as mild strokes [62]. A meta-analysis in 2014, which included nine RCTs (including the NINDS, ECASS, and IST-3 trials), showed that early intravenous rt-PA administration in acute mild stroke (NIHSS 0-4) resulted in a 48% increase in benefit compared to the control [63]. In recent years, clinical studies on tenecteplase have increased. The NOR-TEST trial in Norway, published in 2017, compared the efficacy of 0.4 mg/kg tenecteplase with 0.9 mg/kg rt-PA, revealing comparable effectiveness and safety profiles between the two groups. The majority of enrolled subjects presented with mild stroke (median NIHSS = 4) [64]. Similarly, subgroup analysis of the Canadian AcT trial showed that tenecteplase exhibited comparable efficacy to rt-PA in patients with mild stroke (NIHSS < 8) [65].

Observational studies on thrombolysis for non-disabling mild stroke have yielded positive results. An Austrian national cohort study that enrolled 890 patients with an initial NIHSS of 0-5 found that intravenous rt-PA had a better outcome after 3 months [66]. Similarly, the Chinese National Stroke Registry (CNSR-III), which enrolled 1,905 mild stroke patients (NIHSS 0-5), indicated that IVT independently predicted favorable functional outcomes (mRS 0-1) at discharge, 3 months, 6 months, and 1 year. Subgroup analysis of MaRISS study further showed that IVT was significantly correlated with long-term good outcomes in patients with baseline NIHSS 3-5 [67, 68].

Currently, the only published phase III prospective multicenter RCT, PRISMS, included 313 acute non-disabling mild stroke patients (NIHSS 0-5), comparing IVT with aspirin treatment (156 patients in the thrombolysis group). The results revealed comparable good functional outcomes at 90 days (78.2% vs. 81.5% achieving mRS 0-1), but the thrombolysis group had an increased risk of sICH [69]. This trial was prematurely terminated owing to slow recruitment, failing to meet its target sample size (948 patients), so the reliability of the conclusions is debatable. The ARAMIS trial in China enrolled 760 patients with acute non-disabling mild stroke (NIHSS 0-5), and the results showed that dual antiplatelet therapy was non-inferior to IVT regarding 90-day functional outcomes, with comparable safety profiles [70]. Overall, IVT is recommended in minor disabling AIS in Chinese guidelines, while the effectiveness of IVT in minor, non-disabling AIS remains unclear. Additional research is required to better define the therapeutic value of IVT in these patients.

#### 4.6.2 Elderly Patients

Economic transformations and healthcare advancements during recent decades have substantially enhanced life expectancies across Asian nations [71, 72], precipitating a swift population transition with the proportion of the population aged 60 or more over 40% in several Asian countries by 2050 [United Nations, Department of Economic and Social Affairs, Population Division (2017). World Population Ageing 2017 - Highlights (ST/ESA/SER.A/397)]. As a result, the safety and therapeutic value of IVT in elderly Asian patients remain contentious. A Chinese study found favorable outcomes at both 24 hours and 3 months in 80 years and older AIS patients receiving rt-PA [73]. Conversely, a Korean study observed heightened sICH risk in patients aged 80 and above treated with IVT, though functional outcomes were still improved [74]. Concerning the hemorrhagic complication, a Japanese study from the Fukuoka Stroke Registry employed low dose rt-PA (0.6 mg/kg) in patients over 80 years old within 3 hours of symptom onset finding improved functional outcomes and survival, without significant increases in hemorrhagic complications [75]. These findings highlight the ongoing uncertainty regarding the best treatment strategies for IVT in very elderly Asian patients, underscoring the need for further research in this population.

#### 4.6.3 Anticoagulant Use

Guidelines generally contraindicate IVT in AIS patients previously using oral anticoagulants, while a Chinese cohort study found no significant increase in intracranial hemorrhage, severe bleeding events, or in-hospital mortality after rt-PA treatment in patients using non-vitamin K antagonist oral anticoagulants prior to stroke, compared to those not on anticoagulants [76]. However, studies from non-Asian countries have documented elevated hemorrhagic risks in patients with pre-stroke anticoagulant use [77, 78]. Interestingly, while these studies suggest a higher risk of bleeding, they also indicate that these patients may have comparable or even better recanalization and functional outcomes compared to those without prior antithrombotic use. Notably, recent evidence from Asian populations suggests a reduced incidence of hemorrhage following IVT in patients with prior antithrombotic use compared to non-Asian patients, challenging previous assumptions of elevated bleeding risks in Asians. Given these discrepancies and the guideline contraindication, further investigation is warranted to clarify the safety profiles and clinical outcomes of IVT in this population subgroup with prior anticoagulant use, particularly in Asian populations.

#### 4.6.4 Cerebral Microbleeds (CMBs)

Cerebral microbleeds (CMBs) were not identified as an absolute contraindication for thrombolysis [79]. However, concerns always rise about the risk of hemorrhage in this population, as a higher prevalence of CMBs has been observed in participants of Asian origin[80], potentially due to genetic, environmental, or vascular differences. A previous study of a Chinese cohort receiving intravenous rt-PA demonstrated that extensive CMBs (≥ 3) correlated with elevated rates of parenchymal hemorrhage (PH) at 24h and unfavorable clinical outcomes [81]. However, a Korean study failed to establish a statistically link between CMBs and post-thrombolytic hemorrhagic complications [82]. Similar conflicting findings have been reported in non-Asian populations. A study from a prospective thrombolysis registry in Germany revealed that patients exhibiting multiple CMBs at baseline demonstrated elevated risks of sICH and PH, showing a graded relationship with an increasing number of baselines CMBs [83]. In contrast, a multicenter study in France demonstrated that neither the existence nor the severity of CMBs detected on pre-thrombolysis MRI scans were independently predictive of adverse clinical outcomes or sICH after adjusting for confounding factors [84]. Future RCT are warranted to clarify the relationship between CMBs and thrombolysis outcomes.

## 5. Clinical Research and Guidelines in Intravenous Thrombolysis for Asian Populations

### 5.1 Key Recent Clinical Trials

In recent years, a series of pivotal clinical trials of IVT have been undertaken across Asian populations, providing valuable insights into the safety and efficacy profiles of thrombolytic agents for AIS. Some studies have investigated the efficacy and safety of new thrombolytic agents’ administration. For instance, studies evaluating tenecteplase in China, such as TRACE, TRACE-2, and ORIGINAL, have demonstrated its safety and efficacy within 3 or 4.5 hours of stroke onset. Parallel observations have emerged from India, where tenecteplase exhibits comparable safety profiles and therapeutic efficacy relative to rt-PA. Additionally, research exploring intravenous reteplase in China, such as RAISE, has demonstrated its capacity to yield superior functional outcomes relative to rt-PA when administered within the standard 4.5-hour window. Furthermore, rhPro-UK, a plasminogen activator with a favorable safety profile, has been demonstrated noninferior to rt-PA in multiple trials such as PROST and PROST-2. Other studies have focused specifically on evaluating the administration of thrombolytic drugs beyond the standard therapeutic time window. EXPECTS trial in China extended the therapeutic window for intravenous rt-PA to 24 hours. THAWS trial in Japan extended the window for low-dose rt-PA (0.6 mg/kg) beyond 4.5 h. TRACE-3, ROSE-TNK and CHABLIS-T trials in China extended the window of tenecteplase to 24 hours with imaging guidance. Song et al extending the therapeutic window of rhPro-UK to 6 hours and showing comparable safety and noninferiority. Detailed results are summarized in Table 1.

**Table 1:** Overview of Key Clinical Trials on Intravenous Thrombolysis in the Recent Decade of Asia.

Study	Country	N	Design	Time window	Thrombolytic agent	Dose	Large vessel occlusion confirmation required	Multimodal brain imaging	Primary outcome	Main findings
**EXPECTS, Yan et al. [121]**	China	234	RCT (1:1), alteplase or standard medical treatment	4.5-24 h	alteplase	0.9 mg/kg	No	No	mRS score of 0-2 at 90 days	alteplase higher frequency of functional independence to standard medical treatment with similar sICH
**THAWS, Koga et al. [28]**	Japan	131	RCT (1:1), alteplase or standard medical treatment	>4.5 h	alteplase	0.6 mg/kg	No	DWI-FLAIR mismatch	mRS score of 0-1 at 90 days	similar favorable outcome and safety
**TRACE, Li et al. [42]**	China	240	RCT (1:1:1:1)	0-3 h	tenecteplase, alteplase	tenecteplase: 0.1 mg/kg, 0.25 mg/kg, 0.32 mg/kg; alteplase: 0.9 mg/kg	No	No	sICH within 36 hours	tenecteplase 0.1 mg/kg, 0.25 mg/kg, and 0.32 mg/kg similar safety outcomes to alteplase
Ramakrishnan et al. [122]	India (study I)	50	RCT	0-3 h	tenecteplase	0.1 mg/kg or 0.2 mg/kg	No	No	major neurological improvement at 24h	tenecteplase 0.1mg/kg and 0.2mg/kg similar primary endpoint
	**India (study II)**	91 (India), 144 (NINDS part 1), 168 NINDS part 2)	Tenecteplase 0.2 mg/kg in study I and study II compared with historical controls from NINDS	0-3 h	tenecteplase, alteplase	tenecteplase: 0.2 mg/kg, alteplase: 0.9 mg/kg	No	No	major neurological improvement at 24h	tenecteplase 0.2mg/kg similar primary endpoint to alteplase
**TRACE-2, Wang et al. [43]**	China	1430	RCT (1:1)	0-4.5 h	tenecteplase, alteplase	tenecteplase: 0.25 mg/kg, alteplase: 0.9 mg/kg	No	No	mRS score of 0-1 at 90 days	tenecteplase noninferior to alteplase
**ORIGINAL, Meng et al. [44]**	China	1489	RCT (1:1)	0-4.5 h	tenecteplase, alteplase	tenecteplase: 0.25 mg/kg, alteplase: 0.9 mg/kg	No	No	mRS score of 0-1 at 90 days	tenecteplase noninferior to alteplase, similar safety profile
**TRACE-III, Xiong et al. [30]**	China	516	RCT (1:1) tenecteplase or standard medical treatment	4.5-24 h	tenecteplase	0.25 mg/kg	Yes	salvageable brain tissue identified on perfusion imaging	mRS score of 0-1 at 90 days	tenecteplase less disability and similar survival to standard medical treatment, higher incidence of sICH
**ROSE-TNK, Wang et al. [29]**	China	80	RCT (1:1) tenecteplase or standard medical treatment	4.5-24 h	tenecteplase	0.25 mg/kg	No	DWI-FLAIR mismatch	mRS score of 0-1 at 90 days	tenecteplase safe and feasible
**CHABLIS-T, Cheng et al. [31]**	China	86	RCT (1:1)	4.5-24 h	tenecteplase	0.25 mg/kg or 0.32 mg/kg	Yes	salvageable brain tissue identified on perfusion imaging	major reperfusion without sICH at 24-48 hours	tenecteplase 0.25 mg/kg and 0.32 mg/kg both efficacy and safety
**Li et al. [46]**	China	180	RCT (1:1:1)	0-4.5 h	reteplase, alteplase	reteplase: 12+12 mg, 18+18 mg; alteplase: 0.9 mg/kg	No	No	sICH within 36 hours	reteplase 12+12 mg and 18+18 mg similar safety and efficacy to alteplase
**RAISE, Li et al. [47]**	China	1412	RCT (1:1)	0-4.5 h	reteplase, alteplase	reteplase: 18+18 mg; alteplase: 0.9 mg/kg	No	No	mRS score of 0-1 at 90 days	reteplase more likely to result in an excellent functional outcome than alteplase
**Song et al. [123]**	China	119	RCT (1:1:1)	0-4.5 h	rhPro-UK, alteplase	rhPro-UK: 50 mg, 35 mg; alteplase: 0.9 mg/kg	No	No	mRS score of 0-1 at 90 days	rhPro-UK (50 mg and 35 mg) similar efficacy and safety to alteplase
**PROST, Song et al. [124]**	China	674	RCT (1:1)	0-4.5 h	rhPro-UK, alteplase	rhPro-UK: 35 mg; alteplase: 0.9 mg/kg	No	No	mRS score of 0-1 at 90 days	rhPro-UK noninferior to alteplase
**PROST-2, Li et al. [125]**	China	1552	RCT (1:1)	0-4.5 h	rhPro-UK, alteplase	rhPro-UK: 35 mg; alteplase: 0.9 mg/kg	No	No	mRS score of 0-1 at 90 days	rhPro-UK noninferior to alteplase with similar safety endpoints
**Song et al. [126]**	China	80	RCT (1:1)	4.5-6 h	rhPro-UK	50 mg or 35 mg	No	No	mRS score of 0-1 at 90 days	rhPro-UK (50 mg and 35 mg) effective and safe, no significant differences between different dosages

Abbreviations: DWI: Diffusion Weighted Imaging; EVT: Endovascular Thrombectomy; FLAIR: Fluid-Attenuated Inversion Recovery; mRS: modified Rankin Scale; NIHSS: National Institutes of Health Stroke Scale; RCT: Randomized Controlled Trial; rhPro-UK: Recombinant Human Prourokinase; sICH: Symptomatic Intracerebral Hemorrhage

Moreover, several ongoing clinical trials in China are further exploring the potential application of IVT in extended time windows, new thrombolytic drugs, and cases with contraindications. Notably, the HOPE trial (NCT04879615) aims to investigate whether the time window for intravenous rt-PA can be extended to 24 hours in AIS patients with the presence of potentially salvageable tissue. Other studies, such as EXIT-BT2 (NCT06010628) and TNK-MeVO (NCT06559436), are investigating the feasibility of expanding the therapeutic window for intravenous tenecteplase to 6 hours and 24 hours, respectively. Beyond temporal considerations, researchers are addressing clinical dilemmas of whether thrombolysis should be administered to specific patients through innovative clinical trial designs. The HOPE-BRIDGING (NCT05634382) and TNK-PLUS (NCT062 21371) trials compare clinical outcomes in AIS patients receiving Endovascular Thrombectomy (EVT) with or without IVT beyond 4.5 hours window. Researchers are also examining whether mild stroke patients (NIHSS score ≤5 with specific symptoms) presenting within 4.5 hours of symptom onset could benefit from IVT (ALLOW trial, NCT06115070). Particularly noteworthy are studies tackling traditionally excluded populations with contraindications. The DOAC-IVT (NCT06241677) and I-NOAC trials (NCT06749834) are exploring the efficacy and safety of IVT in AIS patients on direct oral anticoagulants. Similarly, the TIPS trial (NCT06841978) is evaluating IVT in patients with a history of ischemic stroke within the preceding 3 months, while the ICE trial (NCT06841965) explores the efficacy and safety profile of IVT in individuals with concurrent active malignancies.

### 5.2 Development of Clinical Guidelines for Thrombolysis in Asia

According to most Asian guidelines, intravenous rt-PA is widely recommended for AIS patients within 4.5-hour therapeutic window, provided there are no contraindications in China [79], Japan [85], Malaysia [86], India [New Delhi: Ministry of Health and Family Welfare. Guidelines for Prevention and Management of Stroke. 2019. Available at: https://mohfw.gov.in/sites/default/files/Guidelines%20for%20Prevention%20and%20Managment%20of%20Stroke.pdf], and Singapore [Ministry of Health of Singapore. Use of intravenous recombinant tissue plasminogen activator (rtPA) in ischaemic stroke patients. 2013. Available at: MOH_Clinical Guidance_05.indd]. However, Mongolian guidelines limit IVT to 3 hours of symptom onset [Ministry of Health of Mongolia. Guidelines for the management of stroke. 2012. Available at: https://extranet.who.int/ncdccs/Data/MNG_D1_1.%20Clinical%20guideline%20of%20Acute%20Stroke%20.pdf].

Regarding thrombolytic dosing regimens, a consensus exists among Chinese, Indian, Malaysian, and Mongolian guidelines, advocating for an rt-PA administration of 0.9 mg/kg, whereas Japanese guidelines suggest a reduced dosage of 0.6 mg/kg.

For AIS patients with an unknown symptom onset or those presenting between 4.5 and 9 hours, Chinese guidelines recommend multimodal brain imaging (e.g., perfusion-core mismatch identified by CTP or MRP, or DWI-FLAIR mismatch) to identify appropriate candidates for IVT. Similarly, Malaysian guidelines propose extending the thrombolytic window to 9 hours from the last known well state or sleep midpoint, with confirmation of penumbra-core mismatch through CTP or DWI-FLAIR mismatch to estimate a stroke onset within the last 4.5 hours. Japanese guidelines recommend IVT for AIS patients with uncertain symptom onset when DWI-FLAIR mismatch is present. In contrast, extended therapeutic windows are not addressed in Indian and Mongolian guidelines.

In terms of other thrombolytic agents, Chinese guidelines recommend 0.25 mg/kg of tenecteplase for AIS patients within 4.5 hours and urokinase within 6 hours. Malaysia recommends intravenous tenecteplase (0.25 mg/kg) within the 4.5-hour therapeutic window for AIS patients exhibiting large vessel occlusion (LVO) confirmed on imaging. Indian guidelines propose a reduced dosing range of 0.2-0.25 mg/kg tenecteplase within a narrower 3-hour timeframe, whereas Japanese, Singaporean, and Mongolian guidelines do not recommend other thrombolytic agents for IVT.

For patients with mild AIS, Chinese guidelines recommend IVT for those with disabling symptoms within the 4.5-hour therapeutic window, but not for those with non-disabling symptoms. In parallel, Japanese guidelines suggest that IVT could be considered for mild AIS after careful consideration of the indications.

Regarding bridging therapy, Chinese guidelines advise IVT first for patients who meet thrombolytic therapy criteria, with simultaneous consideration of bridging to EVT if appropriate. Malaysian guidelines also recommend thrombolytic therapy prior to EVT in eligible patients presenting within 4.5 hours. Indian guidelines suggest combination IVT and intra-arterial clot extraction if they have internal carotid or proximal middle cerebral artery occlusion causing a disabling neurological deficit. In contrast, Japanese guidelines recommend mechanical thrombectomy without IVT for AIS patients presenting within 4.5 hours of symptom onset who exhibit acute occlusion of the internal carotid artery or the M1 or proximal M2 segments of the middle cerebral artery. Mongolian guidelines, however, do not address bridging therapy. An overview of these guidelines is summarized in Table 2.

**Table 2:** Overview of Clinical Guidelines for Intravenous Thrombolysis in Asia.

Country	Year	Time window	Alteplase dosage	Other thrombolytic agents	Mild AIS	Bridging therapy
**China [79]**	2023	0-4.5 h; For patients with AIS within 4.5-9 hours (including unknown time of onset), if the perfusion-core mismatch identified by CTP or MRP indicates the benefits from vascular recanalisation treatment, IVT is recommended when EVT is not planned or recommended.	0.9 mg/kg	0.25 mg/kg tenecteplase within 4.5 hours; urokinase within 6 hours	For patients with AIS with mild and disabling symptoms within 4.5 hours of onset, IVT is recommended. For patients with AIS with mild non-disabling symptoms within 4.5 hours, IVT is not routinely recommended.	Patients with indications for EVT should undergo treatment as soon as possible. When meeting the criteria for IVT, IVT should be initiated first while simultaneously considering bridging to EVT.
**Japan [85]**	2021	0-4.5 h; If the onset time is unknown, IVT may be considered for patients in whom ischemic changes on DWI are not evident on FLAIR imaging.	0.6 mg/kg	Not recommended at present time because of the lack of sufficient evidence in Japan	Even in patients with mild ischemic stroke, IVT may be reasonable after careful consideration of the indications.	In patients with AIS due to acute occlusion of the internal carotid artery or the M1 segment or the proximal M2 segment middle cerebral artery, EVT without IVT within 4.5 h of onset may be considerable.
**Malaysia [86]**	2021	0-4.5 h; The treatment window can also be extended via CTP with clinical evidence of penumbra-core mismatch up to 9 hours from the time of the last known to be well/midpoint of sleep or via MRI (DWI-FLAIR mismatch) done to identify possible stroke onset within the last 4.5 hours.	0.9 mg/kg	0.25 mg/kg tenecteplase within 4.5 hours with evidence of LVO on imaging	Not mentioned	AIS patients who arrive within 4.5 hours of stroke onset and are eligible for thrombolytic treatment can be considered for intravenous alteplase or tenecteplase prior to EVT.
**India**	2019	0-4.5 h	0.9 mg/kg	0.2-0.25 mg/kg tenecteplase within 3 hours	Not mentioned	Patients with AIS should be considered for combination IVT and intra-arterial clot extraction (using stent retriever and/or aspiration techniques) if they have internal carotid or proximal middle cerebral artery occlusion causing a disabling neurological deficit and patient can be referred to a tertiary healthcare facility, where the procedure can begin (arterial puncture) within 24hours of last known well.
**Singapore**	2013	0-4.5 h; Other means to extend the time window for IVT beyond 4.5 hours using novel agents or MR imaging for patient selection have yet to show efficacy.	Not mentioned	The three rt-PA drugs available in Singapore are alteplase, tenecteplase and reteplase, of which only alteplase has a registered indication for ischaemic stroke thrombolysis.	Not mentioned	Not mentioned
**Mongolia**	2012	0-3 h	0.9 mg/kg	Not mentioned	Not mentioned	Not mentioned

Abbreviations: AIS: Acute Ischemic Stroke; CTP: Computed Tomography Perfusion; MRP: Magnetic Resonance Perfusion; IVT: Intravenous Thrombolysis; EVT: Endovascular Thrombectomy; DWI: Diffusion Weighted Imaging; FLAIR: Fluid-Attenuated Inversion Recovery; LVO: Large Vessel Occlusion; rt-PA: Recombinant Tissue Plasminogen Activator

## 6. Streamlining Processes for Intravenous Thrombolysis in Asia

### 6.1 Discrepancies Between Clinical Guidelines and Real-World Practice

The application of IVT and the door-to-needle time (DNT) in Asian countries tend to be less optimal compared to non-Asian countries. Published studies show considerable variation in DNT and thrombolysis rates across Asian countries. A comparison between AIS patients from the CNSR in China and the GWTG-Stroke in U.S. revealed notable discrepancies, finding that Chinese patients were less frequently to receive thrombolytic therapy (2.5% vs. 8.1%) and experienced longer treatment delays (95 minutes vs. 62 minutes in DNT) compared to their U.S. counterparts [87].

### 6.2 Systemic barriers for application of IVT in Asian countries

Several factors contribute to the differences observed in IVT utilization between Asian and non-Asian countries.

The inadequacy of stroke care resources in many Asian countries is one of the primary influencing factors. According to the latest World Health Organization (WHO) Dementia health and social workforce, the number of neurologists per 100,000 population was 4.38 in Japan, compared to 0.09 in Bangladesh and 0.07 in Myanmar—much lower than in non-Asian countries like Switzerland (13.1) [Organization WH. 2017. The Global Health Observatory. Available at: www.who.int/data/gho/data/indicators/indicator-details/GHO/number-of-neurologists-(per-100-000)].

Infrastructure limits is another systemic barrier in specific Asian countries. The density of stroke diagnostic equipment, such as CT and MRI scanners, is also lower in Asia. According to 2022 WHO Global Atlas of Medical Devices, Southeast Asia has significantly lower numbers of CT and MRI scanners per million population compared to Europe and the Americas. Furthermore, there are significant disparities exist within Asia, with Japan's medical equipment density (111.69 CT and 55.31 MRI per million population) significantly exceeding South-East Asia Region such as Myanmar (0.81 CT and 0.24 MRI per million population) and Bangladesh (0.3 CT and 0.49 MRI per million population) [Organization WH. 2022. Global atlas of medical devices 2022. Available at: https://iris.who.int/handle/10665/364709]. Additionally,a population-based study in rural India reported that only 12% of stroke patients underwent any brain Imaging [88]. Inadequate pre-hospital infrastructure also significantly worsens delays in reaching stroke centers. In numerous Southeast Asian nations, as classified by the WHO, Emergency Medical Services (EMS) remain under-developed [89]. For instance, Nepal faces persistent obstacles such as frequent landslides and basic ambulance services, hindering timely medical assistance. Similarly, India, which shoulders a substantial stroke burden among the Southeast Asian region, lacks a structured EMS framework in the majority of its states and patients had to pay for ambulance transport. Physicians in India reported infrequent implementation of prehospital procedures, including stroke identification (15.7%) and low prehospital notification to hospitals (5%) [90].

Limited financing prevents timely treatment of stroke in many Asian countries, where a lack of insurance systems denies patients access to costly treatments like IVT. Rt-PA can cost up to $1000, which is often unaffordable for patients in low-income countries like Nepal, where the average annual income is $691.7 [91]. Additionally, insurance coverage is often inadequate, as reported by the 2023 Universal Health Coverage (UHC) monitoring report, which shows lower UHC service coverage in South and Southeast Asia compared to North America and Europe [Organization WH. 2023. UHC monitoring report and the executive summary. Available at: https://iris.who.int/bitstream/handle/10665/374059/9789240080379-eng.pdf?sequence=1]. In several lower-middle-income Asian countries, including Vietnam, economic constraints—such as limited healthcare funding and high out-of-pocket expenditures—pose a critical barrier to the equitable adoption of IVT in stroke care [Bank W. 2021. Out-of-pocket expenditure (% of current health expenditure) - Viet Nam. Available at: https://data.worldbank.org/indicator/SH.XPD.OOPC.CH.ZS?end=2021&locations=VN&start=2000&view=chart]. While access to thrombolytic therapy remains a challenge, there are more affordable options such as low-dose rt-PA, which could be promoted to improve thrombolysis rates across Asia. However, Asian countries still face significant hurdles in achieving UHC.

Public education levels and cultural beliefs can also contribute to delays in hospital admission following the onset of stroke. In several Southeast Asian countries, traditional beliefs and treatment are prevalent among the population. Some patients may initially resort to traditional remedies rather than seeking immediate hospital care after the onset of stroke. This delay significantly reduces the efficacy of IVT and compromises treatment outcomes. In rural India, respondents after stroke tend to seek help from private physicians or traditional herbal providers depending upon the accessibility, affordability, and perceived effectiveness of the therapy [92]. Both physicians and the public should be educated about the importance of early referrals and presentations to stroke centers to optimize treatment outcomes.

Interestingly, public awareness of stroke risk factors and warning signs is relatively high across Asia, but knowledge of the importance of timely care and treatment options is lacking, which may hinder timely decisions regarding thrombolytic therapy. Surveys across several Asian countries show that awareness of stroke risk factors ranges from 8% to 87.2% [93, 94] and awareness of warning signs, ranges from 56% to 95.8% [95]. However, when experiencing symptoms, 33% to 53% of individuals chose to call EMS [93, 96]. Public awareness of thrombolysis as a stroke treatment option is lower in Asia, with just 23.3-31% of respondents familiar with thrombolytic therapy for AIS [93, 97]. Awareness of stroke treatment is generally better in non-Asian countries, with 19-92% of individuals familiar with thrombolysis [98, 99]. Interventions to improve knowledge of stroke treatment and encourage appropriate actions after stroke onset are crucial to reducing treatment delays and improving outcomes in Asian populations.

### 6.3 Stroke Center Development in Asia

Stroke centers have been established across Asia to enhance the management and treatment of AIS patients. The concept of stroke centers was first discussed by the Brain Attack Coalition in 2000, which recommended two types of centers: comprehensive stroke centers (CSCs) and primary stroke centers (PSCs). PSCs are designed to deliver standard care for the majority of stroke cases, whereas CSCs provide advanced treatment options and specialized techniques that are beyond the scope of PSCs.

In Asia, stroke center development began in the eastern regions. In 2008, South Korea's Ministry of Health and Welfare (MHW) launched the Regional CSCs program, aiming at reducing stroke incidence and mortality. The implementation of this program led to improvements in key metrics, including faster DNT and shorter hospital stays [100]. Following the success of the Regional CSCs, the MHW expanded the program from 2014 to 2018, focusing on two key objectives: establishing PSCs in rural areas and creating an integrated network linking Regional CSCs with PSCs [100].

In 2011, China's Ministry of Health formed the China Stroke Prevention Project Committee (CSPPC) to enhance stroke care and prevention efforts nationwide. Since 2012, multi-disciplinary stroke center pilot projects have been implemented in major cities like Beijing and Shanghai [101]. In 2015, China issued official guidelines for stroke center construction, recommending both PSCs and CSCs and starting the certification process for stroke center hospitals [102]. To optimize stroke care, the CSPPC implemented a comprehensive network of stroke centers, a stroke mapping system and a "Green Channel" system to facilitate rapid treatment. The goal was to create three critical "1-hour gold rescue circles" for patients: 1 hour from symptom onset to emergency call, 1 hour for pre-hospital transport, and 1 hour for DNT. By July 2023, the National Stroke Center platform reported 2116 registered stroke centers in China, including 613 Advanced Stroke Centers (similar to CSCs) and 1503 Stroke Prevention Centers (similar to PSCs) [101].

Japan's stroke center development is led by the Japanese Stroke Society (JSS), which has been working under the Stroke and Cardiovascular Disease Control Act of 2019. As of 2023, Japan has established a network of stroke centers, divided into two levels: PSCs, which provide basic diagnosis and treatment, and CSCs, which meet higher-level care standards[103]. There were 958 PSCs and 251 CSCs certified by JSS by 2023 [101].

In addition to these three countries, stroke centers have also been established in other parts of Asia, including Kazakhstan, Pakistan, Malaysia, Iran, Vietnam, India, Saudi Arabia, and Qatar. Evidence from several Asian countries indicates that the presence of stroke centers significantly reduces DNT, while improving the utilization rates of IVT and clinical outcomes for AIS patients [104-106].

### 6.4 Quality Improvement in Thrombolysis Treatment Processes in Asia

At the pre-hospital stage, several studies in China have focused on early interventions to optimize stroke care in terms of public education. Stroke recognition program such as the "Stroke 1-2-0" in China has shown association with reduced prehospital delay and increased ambulance arrival rates [107]. In another study, EMS with a prehospital notification procedure led to better outcomes by shortening the onset to needle time (ONT) and improving overall stroke care in Chinese urban areas [108] A Japanese study found that a smartphone-assisted prehospital medical information system, which connects prehospital and in-hospital medical teams through the internet, allowed for rapid analysis of stroke patient data, enabling quicker decision-making [109]. Additionally, prehospital triage scales have been developed to help determine the most appropriate hospital for patients. For instance, a 4-item Stroke Scale was validated as an effective tool for identifying LVO stroke in multi-center settings [110].

At the in-hospital stage, quality improvement initiatives in Korea have focused on the adoption of computerized physician order entry systems to minimize delays from hospital arrival to assessments and IVT administration [111]. Additionally, diagnostic tests and scales have been used to accelerate the evaluation process. For example, the RACE scale is used to detect AIS patients [112]. In China, a quality improvement program comprising ten key interventions has been shown to significantly reduce IVT delays. These interventions encompass forming an acute stroke team, implementing standardized protocols, creating emergency bypass routes, prioritizing CT scans, providing immediate CT interpretation, administering IVT directly on the CT table, and introducing incentive policy [113]. In Korea, the adoption of a standardized script and visual decision aids has effectively shortened DNT by improving patient and family decision-making in a timely and sustainable manner [114].

Several clinical trials and quality improvement initiatives have been conducted in Asian countries to enhance the quality of IVT treatment. One notable study, the MISSION trial conducted in China, employed a cluster-randomized design to evaluate the impact of the PEITEM (Persuasion Environment reconstruction Incentivization Training Education Modeling) intervention delivered via teleconference. The trial revealed that this approach resulted in a modest but clinically meaningful decrease in DNT and improved functional recovery for AIS patients undergoing IVT [115]. A stepped-wedge cluster-randomized trial also carried out in China found that targeted quality improvement interventions, when compared to standard care, led to a higher rate of reperfusion therapy in secondary hospitals [116]. Additionally, a prospective quality improvement project in Shanghai showed that the Shanghai Stroke Service System's intervention improved adherence to stroke care guidelines, which in turn enhanced clinical outcomes [117].

### 6.5 New technologies in stroke care

#### 6.5.1 Artificial Intelligence (AI) in Diagnostics for Stroke Care

AI has emerged as a transformative tool in stroke diagnostics, revolutionizing the speed and precision of stroke identification and clinical decision-making. By harnessing advanced deep learning algorithms, AI can meticulously analyze medical imaging data, including CT and MRI scans, to assist physicians in achieving more accurate and efficient diagnoses of AIS. For instance, AI-assisted diagnostic systems can be integrated into large hospitals, where they can analyze patients' brain imaging data within a short timeframe. These systems could quickly identify and highlight potential ischemic penumbra, significantly reducing diagnostic time and optimizing AIS treatment, including thrombolysis and endovascular therapy [118].

#### 6.5.2 Mobile Health (mHealth) Interventions

Mobile devices, such as smartphones, have become an integral part of our daily lives. The high penetration rate of smartphones in Asia provides a robust foundation for the implementation of mHealth interventions. For instance, specialized mobile applications (apps) or mini-programs can be developed to enable real-time monitoring and health management for populations at high risk of stroke. Additionally, stroke emergency maps can be pre-downloaded (NCT06613074) and accessed on mobile devices, allowing patients to be promptly transported to the most appropriate medical facility during an acute stroke event. Furthermore, mHealth technologies show promising applications in post-stroke rehabilitation, with evidence suggesting that smartphone-based rehabilitation apps have demonstrated potential to assist with stroke recovery [119].

#### 6.5.3 Telemedicine in Stroke Care

Telemedicine has the potential to overcome geographical barriers and aggregate high-quality medical resources, thereby enhancing the effectiveness of stroke patient management. In some Asian countries, such as Japan, the demand for telemedicine is increasing due to an aging population, uneven distribution of medical resources, and shortages in local hospitals. Through telemedicine, elderly patients can complete follow-up consultations at home, reducing unnecessary travel. Patients in remote areas can directly consult with specialists in major cities via video conferencing, gaining access to higher-quality medical services. In China, telemedicine also enables remote rehabilitation guidance for stroke patients, thereby reducing healthcare costs, improving the efficiency of rehabilitation care, and ensuring continuity of rehabilitation treatment [120].

## 7. Challenges and Prospects for Intravenous Thrombolysis in Asian Populations

### 7.1 Challenges in Thrombolysis for Asian Populations

The application of IVT in Asian populations presents several unique limitations and challenges. To advance systemic reperfusion practices for AIS, novel thrombolytic agents are essential, offering either superior efficacy or comparable effectiveness to currently approved therapies in improving patient outcomes. Establishing superiority in subgroups of patients who are currently not suitable for rt-PA or tenecteplase seems to be a reasonable goal. It is also anticipated that the creation of a new generation of thrombolytic medications, featuring improved recanalization rates or reduced adverse effects. These advancements naturally strive for incremental clinical improvements, necessitating rigorous validation via extensive large-sample RCTs.

Public awareness of stroke symptoms and rapid medical intervention continues to be inadequate in many parts of Asia. This lack of awareness delays the recognition of stroke and hinders timely access to thrombolytic therapy. In some developing Asian countries, inadequate healthcare infrastructure represents a significant barrier to the widespread implementation of thrombolytic therapy. A lack of advanced imaging equipment specialized neurological care providers, and well-established stroke centers can hinder timely diagnosis and treatment. The high cost of thrombolytic agents, along with associated medical expenses, presents a financial burden for many patients and their families, further limiting access to effective treatment.

It is important to note that some of the comparisons between Asian and non-Asian populations may rely on studies with variable methodologies, results should be interpreted with caution based on the nature of different studies.

In terms of sample selection, the Asian population was drawn from patients in a single country or region, whereas the non-Asian population was sourced from multiple countries or regions. This introduces heterogeneity and potential representativeness bias in the samples, which may also lead to imbalances in baseline characteristics between the different populations, thereby skewing comparisons of IVT efficacy and complication rates. For instance, if the Asian cohort had a higher proportion of elderly patients or those with comorbidities, this could result in an overestimation of adverse outcomes following IVT. Other reviews have also noted that although the overall proportion of lacunar ischemic stroke in the Chinese population appears to be higher than in Caucasians, differences in study methodologies preclude reliable comparisons.

In the evaluation of IVT efficacy, although most studies utilize common metrics such as the mRS and sICH, differences in the timing of assessments and the professional backgrounds of evaluators may also influence the interpretation of IVT outcomes. Taking the incidence of sICH post IVT as an example, if studies in Asian populations are predominantly retrospective, the underreporting of hemorrhagic events due to incomplete documentation in medical records may lead to an underestimation of sICH rates. In contrast, if studies in non-Asian studies are prospective in design, employ more rigorous monitoring of hemorrhagic events, potentially introducing bias when comparing data between the two groups. This raises questions about the reliability of the conclusion that 'Asian populations have a higher risk of sICH.'

Given these considerations, we also advocate for future research to strive for methodological consistency by establishing standardized research protocols, such as uniform inclusion and exclusion criteria, standardized data collection procedures, and consistent efficacy evaluation metrics. Additionally, promoting more multicenter, multinational collaborative studies can integrate resources across regions, enabling the acquisition of larger and more representative datasets. This approach would enhance the robustness of comparisons between Asian and non-Asian populations.

### 7.2 Future Research Directions and Technological Innovations

The future of IVT will depend on not only improving therapeutic agents and techniques but also refining patient selection and treatment strategies, along with integrating emerging technologies.

#### 7.2.1 Precision Patient Selection for IVT

Accurate patient selection is essential for improving outcomes with IVT. Advances in imaging technologies, such as MRP and CTP, allow for better assessment of brain tissue viability, helping clinicians identify salvageable tissue beyond time-based windows. This personalized approach, incorporating factors such as vascular anatomy and collateral circulation, is particularly relevant for Asian populations, considering their genetic characteristics. As imaging capabilities improve, the potential for extended treatment windows could increase, enabling treatment well beyond the current 4.5-hour window. Medical large model technologies will inevitably refine early screening and risk stratification.

#### 7.2.2 Drug Innovation to Improve Reperfusion and Minimize Complications

While rt-PA remains the standard for IVT, its risk of hemorrhagic complications calls for innovation in thrombolytic therapy. The ideal thrombolytic agent should achieve rapid reperfusion, minimize bleeding risks, feature an extended half-life to allow for single-dose use and re-occlusion prevention, exhibit low allergenic and immunogenic potential, resist plasminogen activator inhibitors, and remain cost-effective. Advanced sequencing and bioinformatics technologies may facilitate the identification of novel thrombolytic compounds. Concurrently, computational modeling and protein engineering techniques, such as domain removal, chimeric protein design, and point mutations, are being utilized to enhance current thrombolytic agents. The encapsulation of thrombolytic drugs, which can be achieved through liposomal or nanoparticle delivery for targeted drug infusion, may be promising. Future drug development may focus on personalized therapies, taking into account genetic profiles and biological markers, to enhance treatment efficacy and reduce side effects.

#### 7.2.3 Optimizing IVT Workflow to Reduce Treatment Delay

The time-to-treatment is critical for the success of IVT. Technologies such as mobile CT and MRI devices allow for immediate imaging upon patient arrival, significantly reducing diagnostic waiting times. AI and machine learning algorithms are streamlining image analysis, enabling quicker identification of thrombus locations and risk tissue, facilitating faster treatment decisions. Additionally, 5G technology can enhance telemedicine, enabling real-time expert consultations, especially in remote areas.

### 7.3 Multidisciplinary perspective on IVT for AIS

Streamlined hospital procedures, supported by multidisciplinary teamwork and automated processes, will further decrease DNT and enhance patient outcomes. From a neurological standpoint, advancements in neuroimaging techniques, such as CTP and MRP, should be integrated to evaluate the application of IVT in extended time windows. Furthermore, attention should be directed toward the efficacy of IVT in specific patients, including those with mild stroke, elderly individuals, novel oral anticoagulants (NOACs) users, and those with ancer comorbidities or recent strokes. The core challenge in emergency settings is the rapid and accurate identification of AIS patients to promptly initiate the thrombolytic procedure and administer IVT. It is crucial to establish collaborative frameworks in emergency medicine, emphasizing seamless coordination among multi-disciplinary teams, including emergency physicians, neurologists, radiologists, and nurses, to ensure the efficient and timely implementation of IVT. Epidemiological insights reveal significant geographic and demographic variations in AIS burden that inform IVT delivery. For instance, studies could investigate the relationships between factors such as race, age, gender, lifestyle and AIS incidence or IVT outcome, thereby providing evidence to support the development of personalized IVT treatment strategies. Furthermore, epidemiological analyses can provide evidence-based policy recommendations regarding the strategic distribution of stroke centers and allocation of healthcare resources, particularly for underserved regions with elevated stroke prevalence. From a Health Economics standpoint, a comprehensive cost-effectiveness analysis of IVT should be conducted, meticulously evaluating both direct costs associated with IVT such as thrombolytic drug expenses, medical equipment utilization, and hospitalization fees—as well as indirect costs, including productivity losses due to illness and family caregiving expenses. Simultaneously, the benefits of IVT therapy should be analyzed, encompassing reduced long-term healthcare expenditures from improved functional recovery, enhanced quality of life, and contributions to societal productivity. Through cost-effectiveness analysis, the economic viability of different IVT treatment strategies can be assessed, providing valuable insights for healthcare policymakers to optimize resource allocation.

The integration of multidisciplinary perspectives facilitates the design of IVT delivery systems that simultaneously achieve clinical accuracy, workflow efficacy, and cost-effectiveness.

### 7.4 Policy recommendations for AIS management

#### 7.4.1 Adjustments to Healthcare Policies

It is recommended to optimize the AIS treatment system by establishing a regionalized stroke center network, enhancing the standardization and unification of stroke emergency management. Additionally, the standardized application of IVT indications should be widely promoted to ensure consistent and evidence-based practices.

#### 7.4.2 Training programs for healthcare workers

Strengthening the training for both neurologists and non-neurologists on the early identification of stroke and the administration of IVT. A professional assessment system should be implemented to evaluate the knowledge and skills of medical staff, thereby improving their diagnostic and treatment capabilities and reducing treatment delays.

#### 7.4.3 New funding models

It is suggested that governments and society invest more resources to improve the accessibility of thrombolytic drugs and upgrade stroke treatment equipment in underdeveloped regions. Additionally, medical subsidies should be provided to reduce the financial burden on patients, thereby increasing the accessibility of IVT treatment.

## 8. Conclusion

In summary, intravenous thrombolytic therapy for AIS has seen notable advancements in Asia, including improvements in drug options and treatment efficacy. Determining the optimal drug for diverse patient populations, improving public awareness about stroke symptoms and timely intervention, and addressing significant infrastructure and economic barriers to access are distinct challenges in Asia. Looking forward, research efforts should prioritize personalized therapeutic strategies, extending the time window through advanced imaging technologies, and bolstering public education on stroke prevention and treatment. Fostering collaboration across disciplines will be crucial to overcoming these challenges and improving treatment outcomes for AIS patients in Asia.
